# IgG3 deficiency extends lifespan and attenuates progression of glomerulonephritis in MRL/*lpr *mice

**DOI:** 10.1186/1745-6150-7-3

**Published:** 2012-01-16

**Authors:** Neil S Greenspan, Myro A Lu, Jacob W Shipley, Xuedong Ding, Qing Li, Dilara Sultana, Maria Kollaros, John R Schreiber, Pingfu Fu, Chaim Putterman, Steven N Emancipator

**Affiliations:** 1 Departments of Pathology, Case Western Reserve University School of Medicine, Cleveland 44106-4943, Ohio, USA; 2 Department of Microbiology and Immunology and the Irving and Ruth Claremon Research Laboratory, Division of Rheumatology, Department of Medicine, Albert Einstein College of Medicine, Bronx 10461, New York, USA; 3 Pediatrics, Case Western Reserve University School of Medicine, Cleveland 44106-4943, Ohio, USA; 4 Biostatistics and Epidemiology, Case Western Reserve University School of Medicine, Cleveland 44106-4943, Ohio, USA; 5 Department of Medicine, Albert Einstein College of Medicine, Bronx 10461, New York, USA; 6 The Pathology and Laboratory Medicine Service, Louis Stokes Cleveland VA Medical Center, Cleveland 44106, Ohio, USA; 7 Floating Hospital for Children at Tufts Medical Center, 800 Washington St., #286, Boston 02111, Massachusetts, USA

## Abstract

**Background:**

Antibodies of the IgG3 subclass have been implicated in the pathogenesis of the spontaneous glomerulonephritis observed in mice of the MRL/MpJ-Tnfrsf6*lpr *(MRL/*lpr*) inbred strain which have been widely studied as a model of systemic lupus erythematosus We have produced IgG3-deficient (-/-) mice with the MRL/*lpr *genetic background to determine whether IgG3 antibodies are necessary for or at least contributory to MRL/*lpr*-associated nephritis.

**Results:**

The gamma3 genotype (+/+ vs. +/- vs. -/-) did not appear to significantly affect serum titers of IgG auto-antibodies specific for double-stranded DNA (dsDNA) or α-actinin. However, while substantial serum titers of IgG3 auto-antibodies specific for double-stranded DNA (dsDNA) or α-actinin were seen in gamma3 +/+ mice, somewhat lower serum titers of these IgG3 auto-antibodies were found in gamma3 +/- mice, and gamma3 -/- mice exhibited baseline concentrations of these auto-antibodies. Analysis of immunoglobulins eluted from snap-frozen kidneys obtained from mice of all three gamma3 genotypes at ~18 weeks of age revealed much higher quantities of IgG in the kidneys from gamma3 +/+ than gamma3 -/- mice, and most IgG eluted from +/+ mice was IgG3. The serum creatinine levels in gamma3 +/+ mice substantially exceeded those of age-matched gamma3 -/- mice after ~21 weeks of age. Histopathological examination of kidneys from mice sacrificed at pre-determined ages also revealed more extensive glomerulosclerosis in gamma3 +/+ or +/- mice than in -/- mice beginning at 21 weeks of age. Survival analysis for IgG3-deficient and IgG3-producing MRL/*lpr *mice revealed that gamma3 -/- mice lived significantly longer (p = 0.0006) than either gamma3 +/- or +/+ mice. Spontaneous death appeared to be due to irreversible renal failure, because > 85% of glomeruli in kidneys from mice that died spontaneously were obliterated by glomerulosclerosis.

**Conclusions:**

The available evidence suggests that IgG3 deficiency partially protects MRL/*lpr *mice against glomerulonephritis-associated morbidity and mortality by slowing or arresting the progression to glomerulosclerosis.

**Reviewers:**

This article was reviewed by Pushpa Pandiyan, Irun Cohen, and Etienne Joly.

## Background

Mice of the MRL/MpJ-*Tnfrsf6lpr *(MRL/*lpr*) inbred strain are genetically predisposed to a spontaneous autoimmune syndrome that resembles human systemic lupus erythematosus (SLE or lupus) in several key features. As in lupus patients, a major cause of morbidity and mortality in both male and female MRL/*lpr *mice is progressive glomerulonephritis [1-3]. The pathogenic cascade that culminates in renal damage in human and mouse has been correlated with the renal deposition of auto-antibodies, many of which exhibit specificity for antigens that derive from cell nuclei [4].

Analysis of antibodies specific for auto-antigens or those deposited in the affected kidneys has revealed that IgG antibodies are the dominant isotype [5]. In terms of IgG subclasses, there is evidence implicating both IgG2a and IgG3 antibodies in the pathogenesis of renal disease in MRL/*lpr *mice [6]. Several distinct experimental manipulations that decreased IgG3 production have been associated with less severe renal disease in mice of the MRL/*lpr *or other inbred strains prone to autoimmune disease [7-9]. Conversely, increased production of IgG3 antibodies associated with expression of the Yaa gene on the MRL background has been correlated with more severe renal disease [7]. Similarly, genetic elimination of IgM secretion in the MRL/*lpr *background was associated with increased titers of serum auto-antibodies of the IgG3 (as well as the IgG1 and IgG2a subclasses) specific for dsDNA and more severe glomerulonephritis and earlier mortality [10]. In all cases, these perturbations likely generated potentially relevant physiologic effects other than altered concentration of IgG3 antibodies in serum. Although passive transfer of substantial amounts of IgG3 monoclonal antibodies with rheumatoid factor activity can elicit renal pathology analogous to the damage that develops spontaneously in MRL/*lpr *mice [11-14], the serum levels of such antibodies required for these effects may surpass those that are physiologically relevant in the spontaneous disease.

In the course of characterizing the J606 myeloma protein, the first identified murine IgG3 antibody, the investigators noted that the J606 Fc regions tended to self-associate at concentrations in the mg/ml range [15]. Subsequent reports revealed that a notable characteristic of many IgG3 antibodies is to self-associate in antigen-independent [16-20] or antigen-dependent [21-24] contexts, the latter at much lower antibody concentrations. We hypothesized that Fc-Fc interaction between IgG3 molecules confers a greater effective valence, and thereby greater functional affinity, permitting antibodies with relatively modest intrinsic (i.e., per site) affinities to bind effectively to highly multivalent antigens [21]. This physical-chemical phenotype, unique among the mouse IgG subclasses, has been rationalized [25] with reference to the remarkable association between IgG3 production and immunization with polysaccharide (or, less commonly, protein) antigens bearing multiple copies of the same epitope [26].

Previous studies with IgG3-deficient mice of the BALB/c background demonstrated that such mice were more susceptible to mortality following pneumococcal infection than wild-type BALB/c mice [27]. These mice were also less effectively protected by a vaccine consisting only of the relevant pneumococcal capsular polysaccharide. Overall, the results were suggestive of a unique role for IgG3 in protecting against encapsulated bacterial pathogens and were consistent with the possibility that cooperative binding (via Fc-Fc interactions) of IgG3 antibodies to bacterial capsular polysaccharides is critical to the mediation of immunity.

Irrespective of the potentially beneficial consequences of antibody self-association, there are reasons to wonder if the unusual physical properties of IgG3 antibodies have any potentially negative consequences *in vivo*. In addition to the circumstantial evidence, recounted above, implicating antibodies of the IgG3 subclass in the pathogenesis of glomerulonephritis in murine models of lupus, antibody aggregation has long been regarded as a possible cause of inflammation and tissue damage [28]. It is of interest, in this context, that IgG3 is typically present at a lower concentration in serum than the other IgG subclasses (IgG1, IgG2a, and IgG2b) [29].

Therefore, to address more definitively the importance of IgG3 antibodies in the spontaneous renal disease of MRL/*lpr *mice, we sought to assess the consequences of genetic deficiency in the capacity to synthesize IgG3 antibodies. In this report, we compare titers of serum auto-antibodies to dsDNA and α-actinin, renal deposition of auto-antibodies, renal function, kidney histopathology, and survival among mice of the three γ3 heavy chain genotypes: +/+, +/-, and -/-.

## Results

### Congenic status of γ3 deficient mice

**Figure 1:**
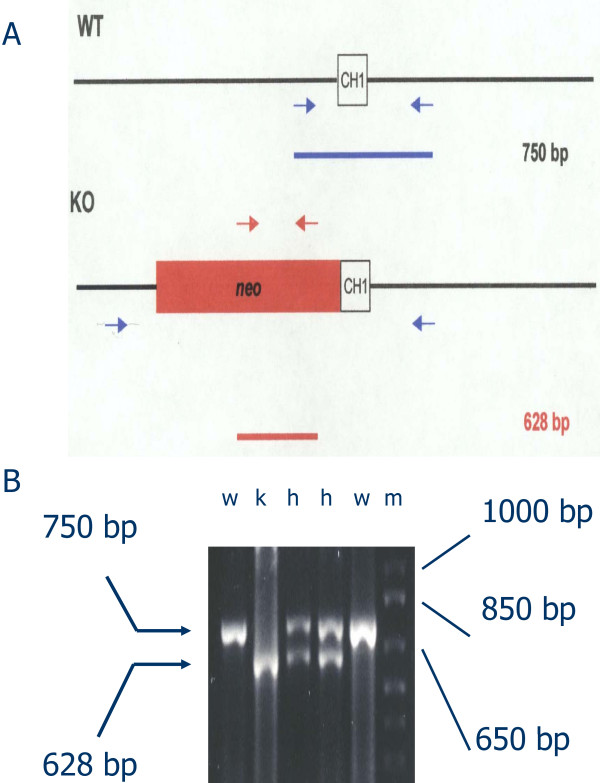
**Polymerase chain reaction (PCR)-based genotyping of representative progeny of a cross between γ3 +/- mice**. A. Schematic of PCR primers used for genotyping: primers for wild-type γ3 allele, blue arrows; primers for defective γ3 allele, red arrows. B. Results for mice representing all three γ3 genotypes (**w**: +/+; **k**: -/-; **h**: +/-). The band produced by the primers for the wild-type (+) γ3 allele is 750 base pairs (bp), and the band produced by the primers for the disrupted (knockout or -) γ3 allele is 628 bp. **m -**size markers.

**Figure 2:**
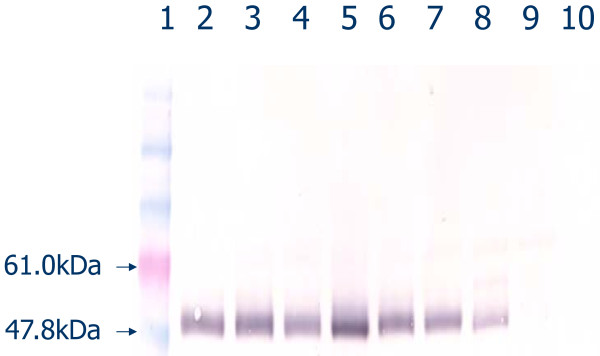
**Western blot for γ3 heavy chain (IgG3) in serum from mice bearing zero (-/-), one (+/-), or two (+/+) wild-type (functional) γ3 heavy chain alleles by PCR genotyping assay**. Lane 1, markers; 2-6, γ3 +/+; 7-8, γ3 +/-; 9-10, γ3 -/-.

IgG3-deficient mice possessing the genetic background of the MRL/MpJ-*Tnfrsf6^lpr ^*(MRL/*lpr*) inbred strain were created through 12 generations of backcrossing mice carrying the defective γ3 heavy chain constant region gene [30] to mice of the MRL/*lpr *strain. Mice of all three γ3 heavy chain constant region genotypes (+/+, +/- and -/-) were generated by crosses between heterozygotes or crosses between γ3 +/- and either -/- or +/+ mice. Figure 1 provides representative genotyping results for the γ3 -/-, +/-, and +/+ mice, and Figure 2 documents the absence of detectable serum IgG3 by immunoblot in γ3 -/- mice.

Two hundred SNPs were typed by single base extension reactions and sequencing by Jax Services (Bar Harbor, ME) for each of six mice typed by us as γ3-deficient. Of the 200 SNPs typed, 199, on chromosomes 1-19, were determined to be of MRL/*lpr *origin for six of six mice (data not shown). One SNP, located on the distal end of chromosome 12 near the γ3 heavy chain constant region locus, was typed as of donor origin in each mouse. A chromosome 12 locus, *sbb2*, identified by Bolland *et al. *[31] as a site for allelic variation that influences autoimmunity, lies approximately 100 Mbp from the targeted Ig heavy chain locus. In all of our tested mice, this *sbb2 *locus is flanked on both sides by SNPs of MRL/*lpr *origin. Similarly, the Fas locus on chromosome 19 is surrounded on both sides by SNPs of MRL/*lpr *origin, strongly suggesting that the γ3 -/- mice have the *lpr *Fas allele.

**Figure 3:**
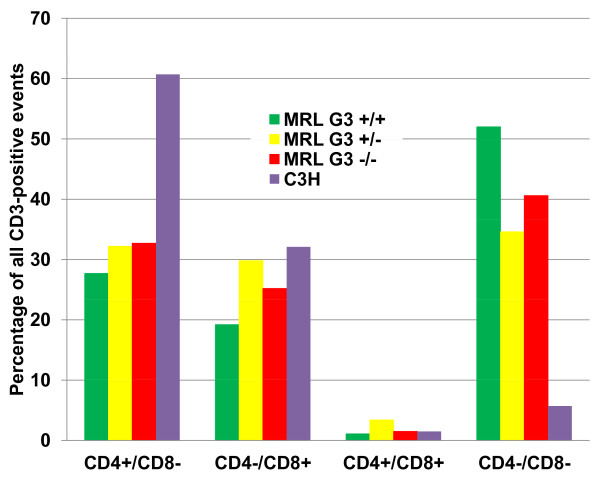
**Relative numbers of T-lymphocyte subsets based on cell surface expression of standard subset markers by flow cytometry applied to spleen cells from MRL/*lpr *mice at 3 months of age and age-matched controls of a strain (C3H) without an obvious predisposition to lupus-like disease**.

Characterization of major T-lymphocyte subsets in the spleen was performed by flow cytometry. This analysis provided an additional test of the success of the backcrossing, as mice of the MRL/*lpr *strain are known to contain significant numbers of cells exhibiting a T cell phenotype (CD3^+^, CD4^-^, CD8^-) ^that is unusual for cells in the periphery in other mouse strains. As revealed in Figure 3, the MRL/*lpr *γ3 -/- mice also have these cells in numbers roughly comparable to the +/+ and +/- mice, consistent with an authentic MRL/*lpr *genetic background.

### Auto-antibodies specific for dsDNA and α-actinin

**Figure 4:**
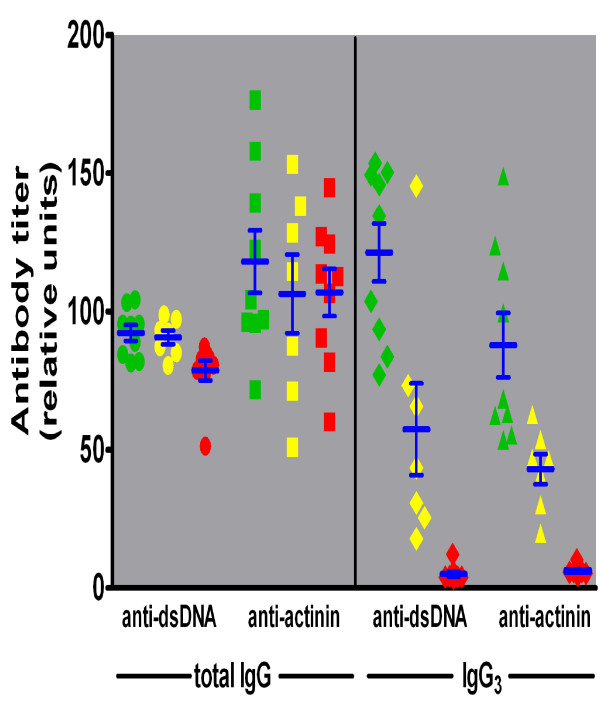
**Serum auto-antibody activity for dsDNA or α-actinin assessed by ELISA**. Serum samples from γ3 +/+ (green), +/- (yellow), and -/- (red) mice were tested for binding of total IgG (left panel) to double stranded (ds) DNA (circles) or to α-actinin (squares), and for binding of IgG3 (right panel) to dsDNA (diamonds) or to α-actinin (triangles).

Titers of serum auto-antibodies with specificity for dsDNA and α-actinin were determined by ELISA using samples from four month-old mice of all three γ3 genotypes (Figure 4). The serum titers of IgG auto-antibodies with specificity for dsDNA were somewhat higher in γ3 +/+ and +/- than in -/- mice (F = 6.0, t ≥ 2.6, p < 0.02), but IgG specific for α-actinin did not differ among the groups statistically. However, there were large and statistically significant ( F ≥ 30.0, t ≥ 3.3, p < 0.01) differences in the serum IgG3 titers for both dsDNA and α-actinin antibodies according to γ3 genotype (Figure 4). These differences establish that whereas significant titers of IgG3 auto-antibodies specific for both dsDNA and α-actinin were present in γ3 +/+ and (to a lesser extent) +/- mice, titers for these antibodies were at baseline levels in the γ3 -/- mice. The greater concentration of the IgG3 auto-antibodies in the IgG3 producing mice could contribute to any observed γ3 genotype-associated differences in renal function, renal histopathology, and survival (see below).

### Elution of immunoglobulin from kidneys

Eluates from the kidneys of γ3 +/+ mice contained more IgG (16.8 or 86 μg/ml at 18 or 21 weeks of age, respectively) than those from -/- mice (6 or 66 μg/mL, respectively). As expected, the IgG3 concentrations in eluates from γ3 +/+ mice far exceeded those in -/- mice (data not shown). Most (> 80%) of the IgG antibodies susceptible to elution from the +/+ kidneys at 21 weeks were IgG3 subclass, and the increased IgG content in renal eluates from +/+ mice at 21 versus 18 weeks of age was almost exclusively of the IgG3 subclass. In contrast, < 12% of the IgG in eluates from -/- mice was IgG3. The eluates from +/- mice were between these extremes, but IgG3 represented only ~10% of the total IgG eluted from +/- mice at either age. Overall, there is considerably more IgG deposited in +/+ than in -/- kidneys, and IgG3 antibodies are a significant fraction of the IgG eluted from the +/+ mice at 21 weeks.

### Renal function over time related to genotype

**Figure 5:**
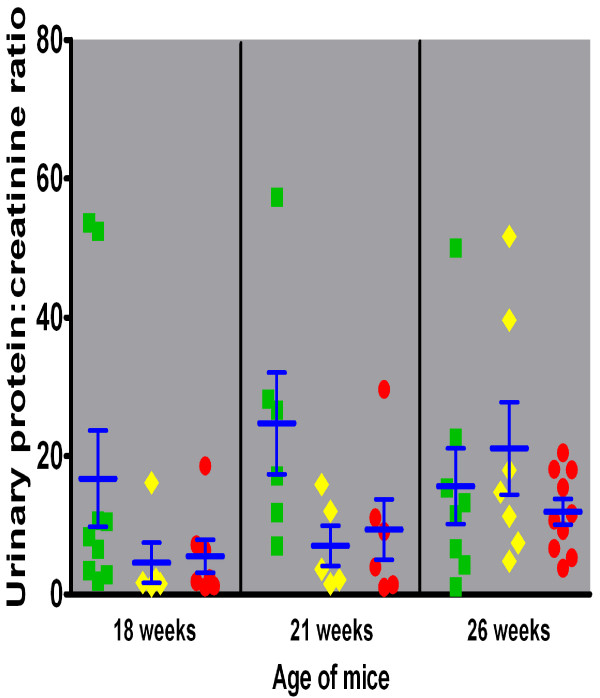
**Urinary protein:creatinine ratio as a function of age in mice of the three MRL/*lpr *γ3 heavy chain genotypes**. All mice, regardless of the γ3 heavy chain genotype, exhibited pathologic and heavy proteinuria throughout the observation period. The mean urinary protein:creatinine ratio exceeded 15 at all time points observed in +/+ mice (green squares), whereas it increased from 4.58 to 21.1 in +/- mice (yellow diamonds) and from 5.5 to 11.9 in -/- mice (red circles).

All mice, regardless of the γ3 genotype, exhibited pathologic and heavy proteinuria throughout the period of timed sacrifices (data not shown). Urinary protein excretion increased from 2.7, 0.8 or 1.7 mg/d in the γ3 +/+, +/- and -/- mice, respectively, at 18 weeks of age to 15.3, 7.4 or 7.6 mg/d in the respective groups at 26 weeks. As a more standardized measure of glomerular permselectivity, the ratio of protein to creatinine in urine exhibited a similar pattern, exceeding 15 in +/+ mice at all times points observed, while increasing from 4.58 to 21.1 in +/- and from 5.5 to 11.9 in -/- mice (Figure 5). The sharp rise in urinary protein:creatinine ratio observed in γ3 +/+ from 18 to 21 weeks and in +/- mice from 21 to 26 weeks was due in part to reduced creatinine excretion, whereas the sustained glomerular filtration in -/- mice (with minor glomerulosclerosis, see below) supported a more gradual increase in the urinary protein:creatinine ratio. In fact, at 26 weeks of age, urinary creatinine was only 0.14 and 0.21 mg/d in +/+ and +/- mice, respectively, but remained at 0.29 mg/d in γ3 -/-, mice. Although the γ3 +/+ mice had a significantly higher protein:creatinine ratio than the +/- or -/- mice at 18 and 21 weeks, the differences among the groups at 26 weeks were not statistically significant. Consonant with diminished urinary creatinine excretion, and presumably a decline in the glomerular filtration rate, urine volume declined in γ3 +/+ and +/- mice, but was sustained at relatively normal values in γ3 -/- mice over the period of timed sacrifice (data not shown).

**Figure 6:**
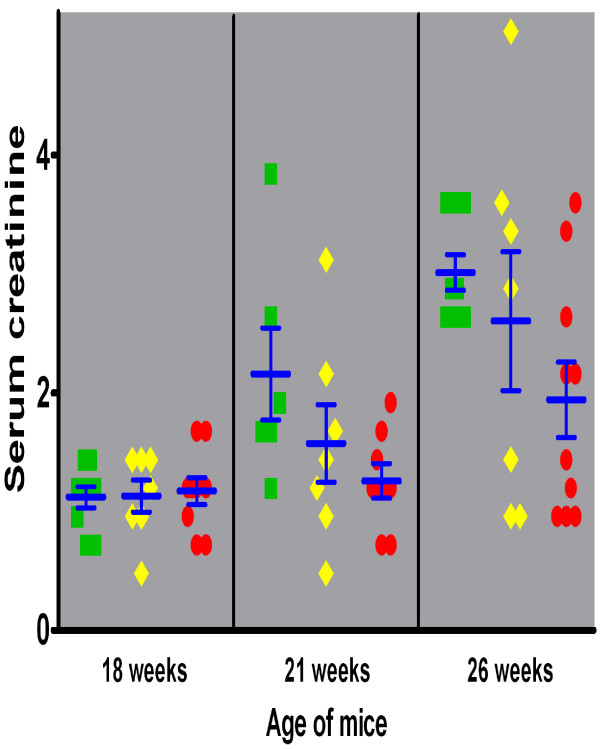
**Serum creatinine as a function of age in mice of the three MRL/*lpr *γ3 heavy chain genotypes**. The serum creatinine increased early (by 21 weeks) during the course of disease in +/+ mice (green squares), whereas -/- mice (red circles) exhibited a more gradual course, with levels of serum creatinine significantly less than in +/+ mice at 21 and 26 weeks. The +/- (yellow diamonds) mice followed an intermediate course.

Perhaps most cogently, the serum creatinine concentration rose sharply and progressively in γ3 +/+ mice over the interval of observation (Figure 6), to a level that was significantly (F = 6.8) higher than that in γ3 -/- mice at 26 weeks (t = 3.02, p < 0.01,). Serum creatinine in both the +/+ and +/- mice at 26 weeks was significantly (t > 3.6, p < 0.01) higher than the level in the same genotype at 18 weeks, whereas the difference in serum creatinine between 18 and 26 week old -/- mice was less pronounced and did not reach statistical significance (t = 2.2). These data indicate that there was progressive renal insufficiency (and presumably nephron loss) in the mice capable of producing IgG3, but this process was attenuated in -/- mice. On the other hand, all three genotypes had similar mean serum creatinine values (of ~1.15 ± 0.11 mg/dl) at 18 weeks of age, which is markedly elevated over normal serum creatinine values (< 0.6 mg/dl) in several different mouse strains. Serum creatinine levels in +/- mice were intermediate between the two homozygous groups, but did not differ statistically from either of the other two genotypes at any time point. Overall, the results are consistent with IgG3 subclass antibodies contributing significantly to the deterioration of renal function over time.

### Renal histopathology over time related to genotype

**Figure 7:**
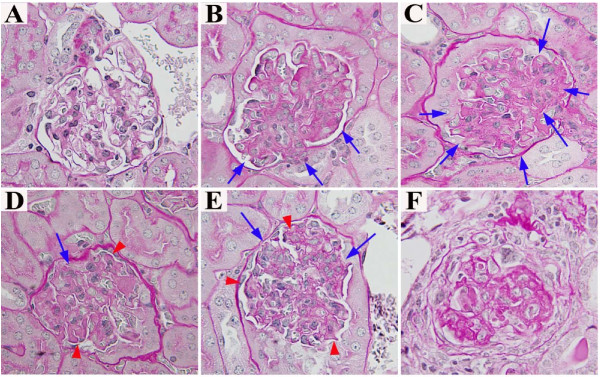
**Representative histopathology for mice of the three MRL/*lpr * γ3 heavy chain genotypes**. At 18 weeks of age, a small minority (~12%) of the glomeruli in each genotype appeared normal (panel A). The most prevalent glomerular pattern (66-76% of glomeruli) at 18 weeks in mice of all three γ3 heavy chain genotypes was segmental (panel B) or global (C) acute endocapillary proliferative glomerulonephritis, wherein some or most glomerular capillaries are narrowed by increased numbers of cells (blue arrows), without changes in the glomerular basement membranes. In γ3 -/- mice at 26 weeks of age, and in γ3 +/+ and +/- mice at 21 weeks of age, chronic endocapillary proliferative glomerulonephritis (panel D, from a γ3 -/- mouse at 26 weeks, and panel E, from a γ3 +/- mouse at 26 weeks) was most prevalent; in these glomeruli, thickening and tortuosity of basement membranes (red arrowheads) is superimposed upon persistently increased endocapillary cellularity (blue arrows). Sclerosis, characterized by collapse, marked thickening and tortuosity of glomerular basement membranes, that occludes capillary lumens and distorts glomerular architecture (panel F), was the most prevalent pattern (67-79%) in glomeruli in +/+ and +/- mice at 26 weeks. In contrast, scarring evident as irreversible glomerulosclerosis was relatively infrequent (≤ 22%) in -/- mice at all time points.

**Figure 8:**
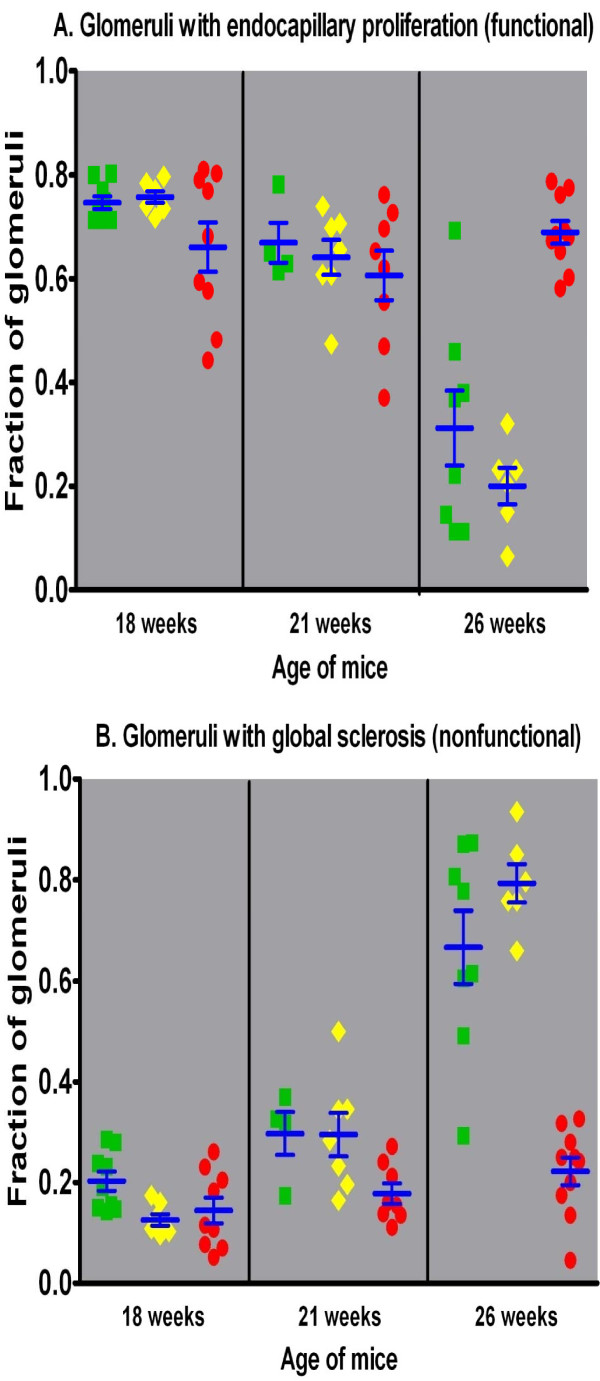
**Morphologic features as a function of age in mice of the three MRL/*lpr *γ3 heavy chain genotypes**. The percentage of renal glomeruli exhibiting either endocapillary proliferative glomerulonephritis (A, top panel) or glomerulosclerosis (B, bottom panel) is plotted as a function of age for mice of each genotype. In +/+ mice (green squares) and +/- mice (yellow diamonds), there is marked loss of functional glomeruli (with endocapillary proliferation) by 26 weeks, with a reciprocal gain in irreversibly scarred glomeruli. In contrast, -/- mice (red circles) exhibit sustained endocapillary proliferative glomerulonephritis as late as 26 weeks, whereas the fraction of scarred glomeruli is only minimally increased.

Histopathologic examination of kidneys from mice of all three γ3 genotypes, sacrificed at selected ages (18, 21, and 26 weeks), also suggests that the absence of IgG3 antibodies influences survival by altering the progression of glomerular damage. Specifically, mice of all three genotypes at 18 weeks of age had a minority of glomeruli with no or only minor mesangial lesions (Figure 7A), and a large majority that exhibited acute endocapillary proliferative glomerulonephritis (Figures 7B and 7C); the frequency of abnormal glomeruli was equal among the genotypes (Figure 8A). By 21 weeks of age, most glomeruli in γ3 +/+ and +/- mice showed chronic changes in glomerular basement membranes superimposed upon sustained endocapillary hypercellularity (Figures 7D and 7E), whereas the γ3 -/- mice exhibited mostly acute lesions, similar to those seen at 18 weeks. At 26 weeks of age, γ3 +/+ and +/- mice progressed to extensive glomerular sclerosis (Figures 7F and 8B). The γ3 -/- mice exhibited a dramatically lower incidence of irreversible destruction of glomeruli (Figure 8B, F = 39.3, p < 0.001).; instead, the majority of the glomeruli in these mice revealed chronic endocapillary hypercellularity (Figures 7D, E, and 8A). Thus, while the γ3 -/- mice clearly suffer renal damage, the process of progression to end stage renal disease appears to be retarded or arrested.

In both human and murine glomerulonephritides associated with lupus or lupus-like disease, glomerular crescents, tubulointerstitial inflammation and interstitial fibrosis are frequent concomitants. A minority of the glomeruli in the mice examined in this work exhibited small crescents, principally cellular at 18 or 21 weeks of age, but mixed fibrocellular or fibrous changes at 21 or 26 weeks of age. The overall frequency and size of the crescents did not differ significantly among the genotypes at any age. Whereas the character of crescents in γ3 +/+ and +/- mice shifted from cellular to increasingly fibrous collaterally with increased age and increased incidence of glomerulosclerosis, fibrous crescents were unusual in γ3 -/- mice, even at 26 weeks old (data not shown). Kidneys from most mice exhibited patchy lymphohistiocytic infiltrates within the tubulointerstitium, and the frequency of such involvement did not differ among genotypes. However, the extent of the interstitial infiltrates and the appearance of tracts of interstitial fibrosis were closely correlated to the extent of glomerulosclerosis, as is typically the case in spontaneous lupus in humans and in other animal models of chronic glomerulonephritis. Therefore, the extent of interstitial inflammation and the extent of interstitial fibrosis were both significantly greater in γ3 +/+ and +/- mice than in the -/- littermates (data not shown).

### Mortality

**Figure 9:**
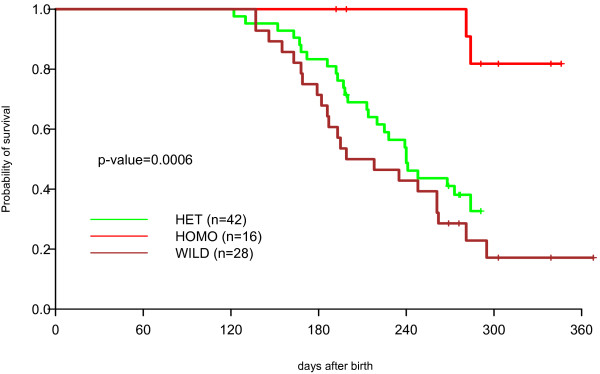
**MRL/*lpr* mouse survival stratified by γ3 heavy chain genotype**. The survival of γ3 -/- mice (HOMO) is statistically significantly greater than the survival of either +/+ (WILD) or +/- (HET) mice.

Analysis of the survival of the first two birth cohorts of MRL/*lpr *mice, including littermates of all genotypes, revealed that γ3 +/+ (n = 28) and +/- (n = 42) mice died at significantly younger ages than γ3 -/- (n = 16) mice (p = 0.0006, Figure 9). Similar patterns of differential survival were observed in both cohorts analyzed separately (p = 0.003 and 0.006, respectively). The survival curves of male versus female mice (of all three genotypes combined) overlap extensively, indicating that gender has no effect on survival (data not shown). The lifespan of γ3 +/+ mice and the lack of a gender effect on survival of the back-crossed mice used herein resemble closely features of the parental MRL/*lpr *strain, and further support the assumption that the mice reported in this communication are of authentic MRL/*lpr *genetic background.

The leading cause of mortality in MRL/*lpr *mice is renal failure [1-3]. In accord with these earlier observations, kidneys removed from γ3 +/+ and +/- mice dying spontaneously revealed extensive (> 85%) glomerulosclerosis. Two of a total of three γ3 -/- mice dying spontaneously were retrieved for histologic examination of the kidneys. One of these had extensive (73%) glomerulosclerosis, whereas the other mice had diffuse endocapillary proliferative glomerulonephritis, with only a minor (28%) extent of glomerulosclerosis.

## Discussion

The conventional interpretation of the pathogenesis of lupus nephritis, in man or mouse, is that IgG auto-antibodies deposit in the kidney in the form of immune complexes, leading to inflammation and tissue damage [4,32]. In the case of lupus-prone mouse strains, substantial circumstantial evidence has implicated antibodies of both the IgG2a and IgG3 subclasses in the pathogenesis of glomerulonephritis leading to renal failure [6-9,11-14,33].

While the evidence implicating IgG3 antibodies in the pathogenesis of the spontaneous glomerulonephritis in MRL/*lpr *mice is substantial, it is not definitive. None of the reported perturbations that affect both the serum levels of IgG3 and the incidence or severity of glomerulonephritis influence only serum IgG3 concentrations. Therefore, the goal of the proposed studies was to define more precisely the role of antibodies of the IgG3 subclass in MRL/*lpr *renal disease by directly comparing IgG3-producing (γ3 +/- and +/+) to IgG3-deficient (γ3 -/-) mice of the MRL/*lpr *background. The current analysis was undertaken with the recognition that even after twelve generations of backcrossing to minimize the chances of cryptic but relevant genetic differences between IgG3-deficient (γ3 -/-) and IgG3-producing (γ3 +/- and +/+) mice of the MRL/*lpr *background, there remains some finite, if low, probability of such allelic disparities [34]. Therefore, to the extent possible, we have compared MRL/*lpr *mice of the three genotypes deriving from the same litters of crosses between γ3 heterozygotes. Genetic analysis of single nucleotide polymorphisms at 200 loci on chromosomes 1-19 (data not shown) strongly suggested that donor origin DNA was restricted to the distal end of chromosome 12 near the γ3 heavy chain constant region locus. Furthermore, the biologic behavior of the γ3 +/+ mice resembled closely that of mice of the parental MRL/*lpr *strain in several characteristic features.

While the presence of two defective γ3 heavy chain constant region alleles did not greatly reduce the titers of auto-antibodies of all IgG subclasses, this genotype was associated with highly significant reductions in IgG3 auto-antibodies specific for dsDNA and α-actinin. Perhaps the most crucial point is that γ3 +/+ mice, and to a lesser extent +/- mice, exhibit substantial serum titers of these IgG3 auto-antibodies that could contribute to progressive kidney damage, whereas in γ3 -/- mice such progression is diminished. If the auto-antibodies of the IgG3 subclass effectively contribute to scarring of kidney tissue, then this difference in the concentrations of these antibodies could account for the observed differences among the mice of the three γ3 heavy chain constant region genotypes. The results from the kidney elution studies are also generally supportive of the importance of IgG3 antibodies in mediating the destruction of functional renal tissue in γ3 +/+ mice.

The mechanism(s) by which IgG3 antibodies might contribute to the pathogenesis of glomerulonephritis were not directly addressed in the present study. Additional investigation will be necessary to determine the relative importance of direct versus indirect pathways in the mediation of tissue damage by IgG3 antibodies. Observations indicating that both IgG2a and IgG3 antibodies are contributory to kidney disease raise the possibility that IgG3 rheumatoid factors (i.e. antibodies able to bind to IgG Fc regions), which can exhibit specificity for IgG2a Fc regions and are present at high frequency in MRL/*lpr *mice [16,17], exacerbate the inflammatory stimulus created by the renal deposition of IgG2a antibodies. The dual presence of IgG2a and IgG3 antibodies in affected kidneys suggests the possible importance of interferon gamma in the pathogenesis of the kidney disease, as this cytokine can enhance production of antibodies of both subclasses [35,36]. Another mechanism that may be operative alone, or in concert with rheumatoid factor activity of IgG3 antibodies, relates to the effects that self-association of IgG3 molecules might exert on effective valence and functional affinity. Increased avidity, or functional affinity, of the antibody component of immune complexes increases the likelihood of complexes to deposit and especially to persist within glomerular capillary walls, independently of other properties of the antibodies or immune complexes [37-39]. In turn, a prolonged dwell time of immune deposits in the glomeruli might promote the increased production of constituents of the basement membrane, leading to more extensive sclerosis and accelerated irreversible destruction of functioning nephrons.

Among mice sacrificed at defined ages, the glomerular pathology in γ3 +/+ and +/- mice evolved from endocapillary proliferation to frank sclerosis far more frequently than in γ3 -/- mice. Histopathologic examination of kidneys recovered from mice dying spontaneously is also consistent with the notion that the absence of IgG3 antibodies attenuates the irreversible renal damage associated with the MRL/*lpr *mouse strain. Nevertheless, it is also clear that even in the absence of IgG3 production, kidney damage ensues, as reflected in endocapillary proliferation.

The comparison of the γ3 +/+, +/- and -/- mice with respect to long-term survival revealed dramatic, and highly statistically significant differences between either +/+ or +/- and -/- mice (p = 0.0006) in the average age of mortality. These results are reasonably, and most simply, interpreted as suggesting that antibodies of the IgG3 subclass contribute significantly to the more rapid mortality of the γ3 +/+ and +/- mice by causing the immune complex-mediated renal disease to progress to end stage renal failure. Given the SNP typing, the nearly identical mortality curves for male and female mice of all γ3 genotypes, and the presence in the γ3 -/- mice of the unusual CD3+, CD4-, CD8- lymphoid population that characterizes MRL/*lpr *+/+ mice, we believe that the γ3 -/- mice have an authentic MRL/*lpr *background, and that it is very unlikely that an unidentified allelic difference between +/+ (or +/-) and -/- mice accounts for the phenotypic differences described herein.

Finally, it is of interest to note that the γ3 heavy chain constant region locus has yet to be associated with the murine lupus phenotype in genetic screening [40-42]. This failure to connect the γ3 heavy chain-bearing antibodies with the disease process may be reflective of a potential, but insufficiently appreciated, weakness of genomic screening approaches. Genome-wide or more focused screens for disease-associated alleles assume that any gene products involved in a pathogenic process will be encoded at polymorphic loci such that some, but not all, allelic products at the relevant locus or loci will be participants in the disease process (or at least will be substantially more contributory than alternative alleles). The γ3 heavy chain constant region gene is the most conserved heavy chain constant region gene in the mouse, and therefore, it may be rendered invisible in gene-disease association studies using standard inbred mouse strains, within which there may be no, or only very limited, polymorphism. Nevertheless, as in the present study, the lack of naturally-occurring disease-influencing polymorphisms at a locus does not prevent the gene product encoded at that locus from contributing to a pathogenic process. This is a particularly important consideration in light of the observations reported in this study, because progression of lupus nephritis, rather than the incidence of the renal disease, varies with the γ3 -/- genotype.

## Conclusions

Antibodies of the IgG3 subclass contribute to the spontaneously occurring glomerular disruption and dysfunction that arises in mice of the inbred MRL/*lpr *strain but are not the sole contributors to this renal tissue damage. In the absence of such antibodies, kidney disease still occurs but, on average, fails to progress to the same extent as in mice able to produce IgG3 antibodies. These results also demonstrate that gene products encoded at loci at which there are no naturally-occurring disease-associated allelic variants can nevertheless contribute significantly to disease pathogenesis.

## Methods

### Genotypes of mice

Experimental animals were maintained in an AAALAC International-approved and specific pathogen-free facility, and used in accord with the protocol approved by the IACUC at Case Western Reserve University. Mice bearing a defective γ3 heavy chain constant region gene on a mixed 129 and C57BL6/J [30] or a BALB/c background [27] have been described. The IgG3-deficient BALB/c mice were used to initiate 12 generations of backcrossing of the defective γ3 heavy chain constant region gene onto the MRL/MpJ-Tnfrsf6lpr (MRL/*lpr*) background. Female and male MRL/*lpr* mice used for the backcrossing were obtained from The Jackson Laboratory (Bar Harbor, ME). Mice of all three γ3 heavy chain constant region genotypes were then bred at Taconic Farms (Germantown, NY) for the present study. Genotyping was done in our lab (Figure 1) using a polymerase chain reaction (PCR)-based assay with the following forward and reverse primers:

1. CH1-up: tcaaacctagctgctaattc

2. CH1-down: tggatatgatcattgacagg

3. NEO 2: cttgggtggagaggctattc

4. NEO 3: caacgctatgtcctgatagc

### Genomic SNP analysis

Tail samples from six MRL/*lpr *γ3 -/- mice were submitted to Jax Services (Bar Harbor, ME) for genomic single nucleotide polymorphism (SNP) typing, with 200 SNPs, by single base extension reactions and sequencing.

### Western blot

Immunoblots for serum IgG3 were performed using samples from MRL/*lpr *γ3 +/+, +/- and -/- mice as described [43]. The IgG3-specific reagent was an alkaline phosphatase-conjugated goat polyclonal antibody (Southern Biotechnology, Birmingham, AL) diluted 1:1,000.

### Flow cytometry

Spleens from 3 month-old MRL/*lpr *γ3 +/+, +/-, and -/- mice and C3H control mice were processed into single-cell suspensions for analysis by flow cytometry. We used the following antibodies to mouse T cells: a cocktail of anti-CD3epsilon-PE-Cy7, anti-CD4-PE, anti-CD8alpha-APC, (BD Biosciences, Sparks, MD). Mouse B cells were identified by anti-CD19-PE (eBioscience, San Diego, CA). A multi-laser flow cytometer, BD LSR (BD Biosciences), was used to analyze about 1 × 10e6 cells per spleen sample. Isotype-matched control antibodies were used in all analyses.

### Measure of auto-antibodies specific for double-stranded (ds) DNA and α-actinin

Concentrations of serum antibodies specific for dsDNA or α-actinin were measured by ELISA as described [44-46]. Briefly, dsDNA (Calbiochem Novabiochem, La Jolla, CA) was purified by filtration through a 0.45-μm filter (Millipore, Bedford, MA) to eliminate contaminating single-stranded DNA, and adsorbed to Immulon II 96-well microtiter plates (Dynatech Laboratories, Chantilly, VA) at a concentration of 100 μg/ml in PBS; plates were dried overnight at 37°C. Before blocking, excess DNA was removed with a 4 min soak in distilled water. Alternately, α-actinin (Sigma-Aldrich, St. Louis, MO) at a concentration of 20 μg/ml was coated onto the same plates overnight at 4°C. Plates were blocked with 3% FCS for 1 h at 37°C and incubated with monoclonal antibodies or serum at a 1/200 dilution for 2 h at RT. Plates were washed five times with PBS-Tween, and alkaline phosphatase-conjugated goat anti-mouse IgG or goat anti-mouse IgG3 (both from Southern Biotechnology Associates, Birmingham, AL) diluted 1/1000 in 3% FCS was added for 1 h at 37°C. Both ELISAs were developed by adding the alkaline phosphatase substrate *p*-nitrophenyl phosphate (Sigma), and the OD was monitored at 405 nm using a MRX Revelation ELISA Reader (DYNEX Technologies, Chantilly, VA).

### Elution of antibodies from kidneys

One-half of a snap-frozen kidney from each mouse was thawed and minced with a clean razor blade. Kidneys from mice of a given cohort were pooled, and Ig was eluted [47]. The eluates were dialyzed at 4°C against distilled water for 24 hours and then against phosphate buffered saline for an additional 24 hours. The amounts of eluted IgG or IgG3 in each group were determined by ELISA, and the antigenic specificities of the eluted antibodies were measured by antigen-specific ELISAs, as described [44,45]. This experiment was performed only once.

### Evaluation of renal function

Randomly selected mice of all three genotypes were sacrificed by exsanguination under ether anesthesia at approximately 18, 21 or 26 weeks of age (within a window of 3 days); serum and kidney tissue were harvested. On the day prior to sacrifice, individual mice were placed into metabolic cages for collection of urine samples over ~16 hours. Proteinuria was quantified by a proprietary protein assay kit (Bio-Rad Laboratories, Hercules, CA) based on a dye-binding procedure [48] in a microplate. The protein concentrations in duplicate urine samples were computed by interpolation of the sample optical densities into a linear calibration curve generated with purified bovine serum albumin (Sigma) over a range of 0 to 1 mg/mL. The creatinine concentration in serum or urine samples was determined by a modified kinetic picric acid binding method [49] adapted for micro-samples as we reported previously [50]. Briefly, alkaline picrate was prepared by adding 5 parts of a proprietary solution of 0.6% picric acid in sodium borate and surfactants (Sigma) to 1 part 1 N NaOH. Next, 20 μL of standard or sample was added to 200 μL of alkaline picrate solution in duplicate microwells. After 10 min at room temperature, the optical density at 490 nm was recorded in a microplate reader. Next, 10 μL of a 2:3 dilution of a proprietary mixture of sulfuric and acetic acids (Sigma) in distilled water was added to each well. Finally, after an additional incubation for 8 min at room temperature, the optical density at 490 nm was again recorded. The difference between the first and second absorbance readings for each sample was used for interpolation into a line correlating the difference in optical density at the two times to standard creatinine concentrations ranging from 0 to 3 mg/dL.

### Evaluation of renal structure

Upon sacrifice (at specific ages selected to provide insight into the progression of the renal disease) or discovery of spontaneous death, each kidney was removed and divided in the transverse plane into three portions. The central portion of each kidney was fixed by immersion in 10% neutral buffered formalin, dehydrated through a graded series of ethanol solutions to anhydrous, washed twice in xylene, and infiltrated with molten paraffin (all reagents from Fisher Scientific, Pittsburgh, PA) in an automated tissue processor (Auto-Technicon, Tarrytown, NY). Tissue blocks were embedded into molten paraffin, sectioned at 2 μm in a Leica microtome, and stained with periodic acid-Schiff reagent with a hematoxylin counterstain. Sections were coded to prevent observer bias, and evaluated by a renal pathologist.

### Survival analysis

During the survival study, mice were observed twice daily, at 7 am and 6 pm; any mice found dead were removed from the appropriate cages. The identifying number of each dead mouse was recorded along with the date and time of death, and kidneys were removed and processed for histological examination as detailed above.

### Statistical analysis

Quantitative data from timed sacrifices (proteinuria, urine creatinine excretion, serum creatinine concentration, and the fraction of glomeruli with each of several histologic features) were evaluated by two-way analysis of variance, considering the genotype and age of each mouse at sacrifice as independent variables. The significance of inter-group variation was evaluated by Bonferroni's t - statistic. Data were pooled from two independent experiments, because three-way analysis of variance disclosed that there was no significant difference between the two replicate experiments.

Overall survival was measured from the date of birth to date of death, and censored at the date of last follow-up for survivors or at the date of sacrifice. (A small number of mice entered into the survival series were sacrificed when moribund for analysis of renal histology.) The differences among groups were tested using the log rank test, and the Cox proportional hazards model [51,52] was used to determine whether genotype is associated with survival after adjusting for any effect of sex. Before applying the Cox model, the proportional hazard assumption was examined. All tests were two-sided and p-values less than 0.05 were regarded as significant.

## List of abbreviations

MRL/*lpr *: MRL/MpJ-Tnfrsf6*lpr*; dsDNA: double-stranded DNA; SNP: single-nucleotide polymorphism;

## Competing interests

The authors declare that they have no competing interests.

## Authors' contributions

All authors read and approved the final manuscript. NG contributed to developing the defective γ3 heavy chain allele, conceived and designed the studies, interpreted the data, and wrote the manuscript. ML harvested tissues and assisted with *in vitro *experiments. JWS harvested tissues and assisted with *in vitro *experiments. XD performed backcrossing and genotyping of mice and *in vitro *experiments. QL performed backcrossing and genotyping of mice and *in vitro *experiments. DS performed *in vitro *experiments. MK performed *in vitro *experiments relating to antibody elution from kidneys. JRS developed the defective γ3 heavy chain allele and bred the original knockout line. PF performed statistical analyses and plotted the survival curves. CP designed and supervised *in vitro *experiments relating to antibody elution from kidneys and wrote the manuscript. SE designed, performed, and interpreted experiments relating to kidney histopathology and wrote the manuscript.

## Reviewers' comments

### Reviewer 1, Pushpa Pandiyan

In the manuscript titled "IgG3 Deficiency Extends Lifespan and Attenuates Progression of Glomerulonephritis in MRL/lpr Mice", Greenspan et al., have studied the role of IgG3 antibody in the autoimmunity associated with MRL/lpr mice. Although IgG3 antibodies are implicated in autoimmunity, their definitive role in causing immunopathology is unclear. These authors have generated IgG3 deficient mice and have shown that the loss of IgG3 protects mice against glomerulonephritis-associated morbidity and mortality. The authors deserve credit for the well-controlled experiments using MRL/lpr mice with all three genotypes derived from the same litters of crosses between heterozygotes. They also deserve credit for presenting their data clearly. The authors have shown that although IgG3 deficient MRL/lpr mice suffer renal damage, the process of progression to end stage renal disease is retarded or arrested, thereby increasing their survival, compared to normal MRL/lpr mice. While these evidence imply that IgG3 antibodies play a pathogenic role in MRL/lpr mice, they are not conclusive. To further strengthen the studies in elucidating the role of IgG3, they should experimentally demonstrate that passive transfer of IgG3 antibodies derived from MRL/lpr mice or injection of hybridomas secreting IgG3 antibodies causes pathology or at least decreases the survival in IgG3 deficient mice.

Minor concerns and questions:

• It is known that IgG3-deficient mice in BALB/c background are more susceptibleto pneumococcal infection than wild-type BALB/c mice. In this study, did the authors observe any infection by commensal bacteria or any evidence of inflammatory bowel disease in IgG3 deficient mice?

• In Figure 1B, the signs " +/+, +/- and -/-" are not aligned with the gel lanes properly.

The manuscript can be accepted for publication in "Biology Direct" provided that the authors perform the suggested experiment.

### Authors' responses to reviewer 1 (Pandiyan)

The experiments requested by the reviewer were done many years ago and reported in multiple publications, two of which are cited as references 13 and 14 in our manuscript.

In the present study, we did not investigate infections or the possible presence of inflammatory bowel disease. Such matters are beyond the scope of the present study.

### Comments on once-revised manuscript - reviewer 1 (Pandiyan)

Antibodies of the IgG3 subclass are previously known to play a role in the pathogenesis of the spontaneous glomerulonephritis in MRL/MpJ-Tnfrsf6lpr (MRL/lpr) mice. However, as put forward by the authors, the rationale of the current study is to provide a direct and a more definitive evidence that IgG3 antibodies are important in the spontaneous renal disease of MRL/lpr mice, using mice with genetic deficiency in the capacity to synthesize IgG3 antibodies. In order to validate the direct pathological role of IgG3, the authors must perform the passive transfer of the antibodies in their mouse model. I agree that it has been previously shown that passive transfer of IgG3 monoclonal antibodies can elicit renal pathology analogous to the damage that develops spontaneously in MRL/lpr mice. However as authors argued, the serum levels of such antibodies required for those effects may surpass those that are physiologically relevant in the spontaneous disease. Moreover those experiments were not performed in mice that were specifically defective in IgG3 subclass antibodies in MRL/lpr background. Therefore, the authors must demonstrate a direct role of this subclass of antibodies in the pathology in mice defective in IgG3 subclass antibodies in MRL/lpr background, which was not done in the previous studies, cited as references 13 and 14. It is necessary to show that such a passive transfer of physiological levels of IgG3 antibodies reverses and reduces the longer life span, observed in the IgG3-deficient MRL/lpr mice in their model. The manuscript can be accepted for publication in "Biology Direct" provided that the authors perform this experiment.

### Authors' responses to reviewer 1 (Pandiyan) regarding initially revised manuscript

While we agree with reviewer 1 that it would potentially be useful to perform passive transfer experiments with IgG3 antibodies in the IgG3-deficient MRL/*lpr *mouse model, such experiments are not guaranteed to recreate the *in vivo *conditions that obtain during the evolution of spontaneous glomerulonephritis. It would take substantial effort to produce the relevant reagents (and we do not know what, if any, antigen specificities these antibodies would need to express), define the necessary experimental parameters (e.g, the amounts of antibodies and the duration of the period of administration), and carry out the experiments. These efforts potentially represent an amount of work sufficient for another manuscript. Furthermore, neither of the other two reviewers commented on the need for passive transfer experiments to justify the publication of the present manuscript. Therefore, we respectfully disagree with the reviewer on this point.

### Reviewer 2, Irun Cohen

The aim of the study was to provide direct evidence that IgG3 isotype antibodies are critical in the glomerulitis that develops in MRL/lpr mice. The method was to backcross a 'defective' gamma3 heavy chain into MRL/lpr mice and observe serum anti-dsDNA antibodies particularly of the IgG3 isotype; kidney lesions; IgG and IgG3 eluted from the kidneys; kidney function and survival. The mice with the 'defective' gamma3 (termed -/-) demonstrated a slight decrease in general serum IgG anti-dsDNA with essentially no IgG3; less IgG and much less IgG3 eluted from the kidneys; modified histologic kidney pathology; and delayed death compared to wild type (termed +/+) and heterozygous mice (termed +/-).

I have several problems with the conclusions one may draw from these results:

1. The so-called -/- mice are transgenic for a defective gamma3 heavy chain; they are not gene knockouts, and, in fact, IgG3 was eluted from their kidneys. Therefore, they do produce at least some IgG3 antibody. So the observations and results cannot be attributed to the absence of IgG3 antibodies.

2. We are not given any details about the actual defect in the transgene, or how expression of the defective gene might have affected expression of the wild type gene that was left, or ideas about the possible role of IgG3 in the recognition of dsDNA, or the likely relationship between the mouse IgG3 isotype and the equivalent human IgG isotype (IgG2?) and their possible roles in SLE in humans vs mice.

3. The mice with the defective IgG3 heavy chain gene died, albeit later than their counterparts; was there any effect of the transgene on the general lymphoproliferative disease of the mice? Did the transgene affect only the kidneys? What actually killed the mice? I enjoyed reading the paper; I found it to be thought provoking and raised the issue of how a single gene product might affect the pathophysiology of a complex immune system and organ system disease.

### Authors' responses to reviewer 2 (Cohen)

The original gamma 3 heavy chain-deficient mice were obtained by homologous recombination of the targeting vector at the gamma 3 heavy chain locus (ref. 30 in the manuscript). The SNP analysis, included for reviewers only, strongly suggests that the defective gamma 3 heavy chain allele inserted as intended eliminating the wild-type allele and making the genetically-manipulated mice true knockouts.

The targeting vector, as described in ref. 30, contained a neomycin-resistance gene just upstream of the sequence coding for C_H_1. This gene structure was designed to be unexpressed.

The Western blot analysis of sera from mice possessing the defective gamma 3 heavy chain allele in both ref. 27 and the present manuscript support the inference that if there is any IgG3 antibody in the sera of these mice it is at a concentration below the level of detection of this assay. In addition, in ref. 27 it was shown by ELISPOT analysis that there were no spleen cells spontaneously secreting IgG3 (sensitivity approximately one cell per million) in mice of the BALB/c background that were genotyped as gamma 3 -/-. In contrast, spleens from wild-type BALB/c mice contained numerous cells secreting IgG3.

Mouse IgG3 and human IgG2 are not fully 'equivalent,' although both isotypes are associated with responses to polysaccharide immunogens. Human IgG2 has not been described to dynamically self-associate non-covalently like murine IgG3, but Yoo *et al. *(J Immunol. 2003 Mar 15;170(6):3134-8. PubMed PMID: 12626570) have described IgG2 dimers that form due to disulfide bonds between Fc regions of otherwise separate IgG monomers.

### Reviewer 3, Etienne Joly

The manuscript by Greenspan et al. describes that, when an inactivated IgG3 locus is introgressed in MLR/lpr mice those become partially protected against glomerulomephritis.

On the positive side, I found the observations reported convincing, and those could clearly be relevant to deciphering the pathogenesis of kidney damage in lupus, and other antibody-mediated diseases.

On the other hand, although the overall quality of the English was more than adequate, I also found that many aspects of the work were very poorly presented, and this was particularly true for the material and methods and the results sections. I would therefore strongly suggest that many modifications should be made to the manuscript before it is published. Below, I have provided a non exhaustive list of suggestions, but I would also advise the authors to seek advice from willing colleagues inside and outside of their immediate field of study to help them improve the overall structure and contents of their manuscript.

1) Nowhere in the title or abstract does it say that MRL/lpr mice are a commonly used model for SLE. In fact, I would also suggest adding a bit more info on these mice in the introduction, such as that provided at the Jackson (http://www.informatics.jax.org/external/festing/mouse/docs/MRL.shtml)

2) In the abstract, I suggest replacing the sentence "gamma3 -/- mice exhibited minimal titers of these auto-antibodies " by "gamma3 -/- mice exhibited baseline levels of these auto-antibodies"; Indeed, 'minimal titers' suggests that there are some IgG3 still present, and according to McLay et al. 2002, the IgG3 KO mice have a complete absence of IgG3 synthesis. Any signal detected should be due either to background noise, or cross reactivity of the reagents on other isotypes.

3) In the introduction, in the sentence "In terms of IgG subclasses, there is evidence implicating both IgG2a and IgG3 antibodies in the pathogenesis of renal disease [6]" you should specify whether this is true for humans, mice, or both

4) further down, I would change 'Subsequent reports revealed that many IgG3 antibodies self-associate in antigen-independent [16-20]' to 'Subsequent reports revealed that a specific particularity of many IgG3 antibodies is to self-associate in antigen-independent [16-20]'

5) In the section on genotypes of mice in the M&M, there should be a much more thorough description of the IgG3 +/- genotyping protocol, with a reference to panel A of Figure 1. In fact, it may be best to describe the whole of Figure 1 in the M&M. The SNP genotyping should then be presented as a separate paragraph (or section even) Since the Fas locus is very important in the MRL/lpr phenotype, it would also be good to underline that this region is indeed correctly from the MRL origin. There is also a typo on the last line: table, not tabel.

6) In the section entitled, Measure of auto-antibodies specific for double-stranded (ds) DNA and g-actinin, I am not exactly sure of what the purpose of the passage of dsDNA through a 0,45 micron filter is. It is possibly to sterilize it, and/or to shear the DNA, but certainly not to purify it !

7) In the section "Elution of antibodies from kidneys": the buffer(s) used, and the precise conditions should be specified ( volumes, temperature, time, shaking or not...). Also, I suggest changing 'The total eluted IgG or IgG3 in each group was determined ...' to 'The amounts of eluted IgG or IgG3 in each group were determined ...'. On this same subject, in the results section, the amounts of antibodies eluted are expressed as concentrations, which really does not mean anything if one does not know the final volume used for elution. I would strongly suggest expressing those as total amounts of antibody per kidney, or per mouse.

8) Evaluation of renal structure: Upon sacrifice (at specific ages selected to provide insight into ( not to) the progression

9) I suggest that the first paragraph of the results really belongs in the M&M section. The layout of Figure 1 has also gone wrong( lane legends are nto aligned with picture), but I suspect this has happened during the submission process. As a whole, however, I found that the layout of figures was really awkward, and not bearing in mind that those would be printed as a very small fraction of a page in the final pdf. Blank areas should thus be kept to a minimum, and important information presented in a way that can sustain shrinking (such as the picture of the gel on Figure 1)

10) On Figure 3, I find it really bizarre not to have the percentages of normal Balb/C mice. As it is, how do we know that the low percentage of DN is not an effect of the IgG3-/- specific to the Balb/c background?

11) In the text describing Figure 4, I would replace 'minimal levels' by 'baseline levels'. The last sentence of that sections is completely out of place here: you have not made any previous mention of differences in renal function in the -/- mice yet !!

12) In the section on eluted antibodies: as mentioned earlier, these need to be expressed as total amounts (in micrograms) per kidney or mouse. You also need to describe how the ratios between total IgG and IgG3 were established. The most disturbing thing to me, however, was that these data were not presented as a figure. Is this because this was done only once or twice (which would explain why there is not deviation provided)? If it is the case, you should really repeat this a few more times instead of providing data in this shoddy format which is both difficult to apprehend, and does not correspond to data which had been documented sufficiently thoroughly.

13) Regarding figure plots, I would strongly encourage you to convert them to a smaller format with white backgrounds. Figures 4, 5, 6 and the data on eluted antibodies could then all be presented on a single figure with 4 small panels.

14) I would like to express my very strong disagreement (not to say outrage) regarding the following sentences: ""Serum creatinine in both the +/+ and +/- mice at 26 weeks was significantly (t > 3.6, p < 0.01) higher than the level in the same genotype at 18 weeks, whereas the difference in serum creatinine between 18 and 26 week old -/- mice was not significant (t = 2.2). These data indicate that there was progressive renal insufficiency (and presumably nephron loss) in the mice capable of producing IgG3, but not in -/- mice.". Indeed, just because a difference does not reach statistical significance, it does not mean that there is no difference. I would thus suggest the following wording: "Serum creatinine in both the +/+ and +/- mice at 26 weeks was significantly (t > 3.6, p < 0.01) higher than the level in the same genotype at 18 weeks, whereas the difference in serum creatinine between 18 and 26 week old -/- mice was less pronounced and did not reach statistical significance (t = 2.2). These data thus suggest that there was progressive renal insufficiency (and presumably nephron loss) in the mice capable of producing IgG3, but that this was attenuated in -/- mice."

15) I found the whole section on histopathology extremely difficult to read and understand. Indeed, whereas, in other sections of the manuscript a clear effort is made to be didactic for a non specialist reader ( which is commendable since Biology Direct is not a specialty journal), I found that this section was incredibly difficult to decipher. I would actually suggest pooling Figures 7 and 8, and placing the actual titles next to each of the histology panels rather than letters, to make it clear that those are provided as examples, and not representative of mice of one or another genotype. And once the manuscript has been modified, I would strongly urge you to seek the assistance of colleagues outside the field to help make this section into something that is more easy to understand for people like me.

16) Regarding Figure 9, it took me a while to convince myself that the data for all three groups of mice had been pooled. This is simply not an appropriate procedure ! I suspect that, once again, this was due to the fact that you did not have sufficient numbers of mice in each group to plot the survival curves independently for each group, and looking at the curves, it seems to have been due to a lack of females, most probably because those had been needed to breed to maintain the colony and generate more mice. In this kind of situation, where you have made the observation over and over again, but do not have the right numbers of mice in each group, I would suggest to take Figure 9B out, and simply stating in the text that, as in the MRL/lpr parental strain, you did not detect any gender effect in any of the backcrossed groups.

17) In the discussion, the idea about an IgG3 rheumatoid factor is interesting, but it would be helpful to define clearly what a rheumatoid factor is (i.e. an antibody that can bind to the Fc portion of other antibodies).

18) Further down, replace "with the notion that elimination of IgG3 antibodies attenuates the irreversible renal damage associated with the MRL/lpr mouse strain." by "with the notion that the absence of IgG3 antibodies attenuates the irreversible renal damage associated with the MRL/lpr mouse strain."

### Authors' responses to reviewer 3 (Joly)

1) The abstract now notes that the MRL/*lpr* strain has been widely studied as a model of human systemic lupus erythematosus.

2) The requested wording change in the abstract has been implemented.

3) The relevant sentence (p. 4) now indicates that the reference to Ig3 and IgG2a antibodies applies to mice. Humans do not have a subclass referred to as "IgG2a."

4) The relevant passage (Introduction, paragraph 3) has been modified to read as follows: "Subsequent reports revealed that a notable characteristic of many IgG3 antibodies is to self-associate in antigen-independent [16-20] or antigen-dependent [21-24] contexts."

5) In the M&M section on "Genotypes of mice," reference is now made to Figure 1. As suggested, we have also moved the sentences about the SNP typing to a separate section with a sub-heading, and the implications of the SNP analysis for the allelic origins of the genes at the Fas locus on chromosome 19 are now explicitly noted in the appropriate subsection in the Results (p. 9). The typographical error in the last line of what is now the "Genomic SNP analysis" section has been fixed.

6) Passage through the 0.45 micron filter is to remove the single stranded DNA, thus enriching for dsDNA. This is a standard method which has been used in all of our (CP) previous relevant publications, and it has never been questioned.

7) The wording changes requested have been implemented in the M&M section on the methods for elution of IgG antibodies from kidneys. As requested, details of the methods have also been inserted.

8) The requested revision in the text has been implemented in the M&M section on "Evaluation of renal structure."

9) For the gel photograph in Figure 1, we have substituted single-letter abbreviations (w - wild-type; k - knockout; h - heterozygote; m - markers) which we hope will better preserve alignment with the correct lanes throughout the submission and publication process.

10) We have reduced Figure 3 to one panel focused on T-cell subsets. The data presented are from an experiment in which spleen cells from wild-type C3H mice were used for comparison to the spleen cells from MRL/*lpr *mice of all three γ3 genotypes (+/+, +/-, -/-). Similar results were obtained in a repeat experiment with spleen cells from BALB/c γ3 -/- mice.

11) The requested revision has been made (Results, paragraph 4; "minimal levels" replaced by "baseline levels"). In addition, we have modified the wording of the final sentence of the section to read: "The greater concentration of the IgG3 auto-antibodies in the IgG3 producing mice could contribute to any observed γ3 genotype-associated differences in renal function, renal histopathology, and survival (see below)."

12) See response to comment 7.

13) We acknowledge the reviewer's comment but do not believe it is necessary to consolidate the figures or create a figure for the limited kidney elution data.

14) We have implemented requested change in wording in paragraph 7 of Results.

15) As suggested, the first paragraph of the section has been significantly revised.

16) As suggested, we have eliminated Figure 9B and now refer to the corresponding results in the text.

17) A description of rheumatoid factors has been inserted, as requested, in paragraph 4 of the Discussion.

18) We have replaced "elimination" with "the absence" in paragraph 5 of the Discussion.

### Comments on once-revised manuscript - reviewer 3 (Joly)

The manuscript by Greenspan et al. describes that, when an inactivated IgG3 locus is introgressed in MLR/lpr mice those become partially protected against glomerulomephritis. I found the observations reported convincing, and those could clearly be relevant to deciphering the pathogenesis of kidney damage in lupus, and other antibody-mediated diseases.

Compared to the initial version, I find that the modifications introduced following the suggestions of the three referees have resulted in a very significant improvement of the manuscript.

I do, however, remain thoroughly unsatisfied with the aspects dealing with elution of immunoglobulins from kidneys, for the following reasons:

- The authors only provide the concentrations of antibodies recovered, but not the total volumes of those eluates. There is not even an indication that the same volumes were used for all samples. What should really be provided, and compared, are amounts of antibodies per kidney (or per mg of tissue).

- From the way the data is presented in the text, I have come to suspect very strongly that these data were obtained only once, with each value corresponding to one eluate obtained from the pooled half-kidneys in each of the cohorts. I can understand that this may be an unavoidable limitation of the approach, but this should have been made very clear, and justified, in the text.

- In this paragraph, the authors state that "< 12% of the IgG in eluates from -/- mice was IgG3.", suggesting that there are some IgG3 to be found in IgG3 KO mice. According to McLay et al. 2002 (ref 27), however, the IgG3 KO mice have a complete absence of IgG3 synthesis. Any signal detected would thus be due either to background noise, or cross reactivity of the reagents on other isotypes.

- And what to say of the +/- mice, for which 'IgG3 represented ~10% of the total IgG eluted', i.e. roughly the same levels as those from IgG3 KO mice?

- All those considerations are particularly relevant since, contrary to what the authors state, the differences in total IgG concentrations between +/+ and -/- mice are actually rather minor: 16.8 vs 6 μg/ml at 18 weeks, and 86 vs 66 μg/ml at 21 weeks. From what I can make out from the data in the form in which it is provided, the IgG deposits are mostly of the IgG3 subtype in +/+ mice, and not so much less, but of other subtypes in -/- mice, and very surprisingly also in +/- mice. This last point is completely brushed over by the authors. This observation in itself underlines the need for those experiments to be repeated on additional mice, or cohorts of mice, before those results can be considered as sound and reliable.

I was also sorry to see that the authors had chosen to ignore my suggestions on two much less important points:

- In the paragraph of the M&M section about anti dsDNA ELISA, I thought that it would be helpful to clarify that the purpose of passing the DNA through a 0.45 micron filter was to eliminate single stranded DNA.

- I also suggested that the layout of figures should be modified. As they are now, I suspect that those figures will become extremely difficult to read once they have been reduced to printing sizes, and will become completely useless to people if printed in black and white (as well as for color-blind people).

### Authors' responses to reviewer 3 (Joly) regarding initially revised manuscript

We now explicitly state in the relevant section of Materials and Methods that the kidney elutions were only performed once. As for the methodology, the identical volumes of fluid were used for eluting each kidney. We believe that the apparent IgG3 concentrations eluted from the γ3 -/- kidneys most likely arose from the crossreactivity of the detection reagent for the ELISA.

As requested, we have added the rationale for the filtration of the DNA to the section on the ELISA for autoantibodies specific for dsDNA in Materials and Methods.

Although we appreciate the reviewer's substantial efforts in reviewing the manuscript and take seriously his suggestions (as demonstrated by implementing almost all of them), we are not convinced that it will be a net benefit to modify the figures.
